# A systematic review of using and reporting survival analyses in acute lymphoblastic leukemia literature

**DOI:** 10.1186/s12878-016-0055-7

**Published:** 2016-06-08

**Authors:** Chatree Chai-Adisaksopha, Alfonso Iorio, Christopher Hillis, Wendy Lim, Mark Crowther

**Affiliations:** Departments of Clinical Epidemiology and Biostatistics, McMaster University, Hamilton, Canada; Departments of Medicine, McMaster University, Hamilton, Canada; Departments of Oncology, McMaster University, Hamilton, Canada

**Keywords:** Acute lymphoblastic leukemia, Mortality, Systematic review, Regression analysis

## Abstract

**Backgrounds:**

Survival analysis is commonly used to determine the treatment effect among acute lymphoblastic leukemia (ALL) patients who undergo allogeneic stem cell transplantation (allo-SCT) or other treatments. The aim of this study was to evaluate the use and reporting of survival analyses in these articles.

**Methods:**

We performed a systematic review by searching the MEDLINE, EMBASE and Cochrane library databases from inception to April 2015. Clinical trials of patients with ALL comparing allo-SCT compared to another treatment were included. We included only studies that used survival analysis as a part of the statistical methods.

**Results:**

There were 14 studies included in the review. Sample size estimation was described in 4 (29 %) studies. Only 4 (29 %) studies reported the list of covariates assessed in the Cox regression and 6 (43 %) studies provided a description of censorship. All studies reported survival curves using the Kaplan-Meier method. The comparisons between groups were investigated using the log-rank test and Wilcoxon test. Crossing survival curves were observed in 11(79 %) studies. The Cox regression model was incorporated in 10 (71 %) studies. None of the studies assessed the Cox proportional hazards assumption or goodness-of-fit.

**Conclusions:**

The use and reporting of survival analysis in adult ALL patients undergoing allo-SCT have significant limitations. Notably, the finding of crossing survival curves was common and none of the studies assessed for the proportional hazards assumption. We encourage authors, reviewers and editors to improve the quality of the use and reporting of survival analysis in the hematology literature.

## Background

Survival analysis measures the time from a defined starting point to the occurrence of an interested event where the risk changes over time. The goals of survival analysis serve three purposes: (1) to estimate survival and hazard functions from survival data, (2) to compare survival and hazard functions between groups and (3) to assess the relationship between predictor variables and survival time. The essential components for survival analysis include the time to event and the binary event outcome (success or failure).

The probability of survival can be represented generating a Kaplan-Meier (KM) curve from survival data. Indeed, the KM plot is based on the estimate of the conditional probability of the time to failure [1] calculated at each time point recording an event. The difference in survival between two or more groups (or the treatment effect if treatment is what defines the two groups) can be commonly compared using the log-rank test [2].

The Cox proportional hazard (PH) model is a widely used regression method for survival data. The Cox PH model estimates the effect of predictor variables using the hazard function which does not require specifying a baseline hazard rate [3]. The measure of the effect, unadjusted or adjusted for covariates, is demonstrated as a hazard ratio (HR) which is expressed as an exponent of a regression coefficient in the model. An important property of the Cox PH model is that the PH assumption requires the hazard ratio to be constant over time [4]. Therefore, the Cox PH model is considered to be a semi-parametric model. Other regression models that can be used for survival analysis include an extended Cox PH model or parametric survival model (Weibull, exponential, log-logistic, lognormal, etc.) [5].

A recent systematic review demonstrated that survival analysis was incorporated in only 29 % of internal medicine articles [6]. However, there has been an increasing trend to using survival analysis in all categories of medical journals [6].

Allogeneic stem cell transplantation (allo-SCT) is the most potent post-remission therapy in adult acute lymphoblastic leukemia (ALL). The benefit of allo-SCT in adult ALL remains controversial [7]. Survival analysis is generally used to determine the treatment effect among ALL patients who undergo allo-SCT or other treatments, both in terms of prolongation and increased likelihood of survival. Allo-SCT is associated with high treatment-related mortality. Patients who tolerate the treatment are more likely to have a prolong event free survival and overall survival. On the other hand, non allo-SCT is less intensive treatment but may be associated with lower long-term event free survival. ALL literature were chosen because we expected that the use and report of survival analysis in such articles are complicated. To investigate whether the heterogeneity in study results is at least in part explained by a more or less appropriate use of time to event analysis, we conducted a systematic review of clinical trials which investigated the efficacy and safety of allo-SCT in adult patients with acute ALL. The aim of this study was to evaluate the use and reporting of survival analyses in these articles.

## Methods

### Data sources

We performed a systematic review by searching in the MEDLINE, EMBASE and The Cochrane library (The Cochrane Register of Controlled Trials and Cochrane Database of Systematic Reviews) databases. The reference lists were searched from the retrieved articles. The search terms were: Bone Marrow Transplantation OR Hematopoietic Stem Cell Transplantation OR Peripheral Blood Stem Cell Transplantation AND nonmyeloblat* OR non-myeloblat* OR Precursor Cell Lymphoblastic Leukemia-Lymphoma OR lymphoblast* OR lymphoid. AND (random* OR RCT OR control* OR trial). The database search was performed from inception to April 2015 with no language restrictions.

### Selection criteria

The studies were included if they met the following criteria; were a clinical trial, controlled clinical trial or randomized control trial with allo-SCT compared to autologous SCT or non-transplantation therapy in patients with ALL in first complete remission. We only included studies that used survival analysis as one of the statistical methods.

### Study selection and data extraction

Two investigators (CC and CH) independently identified articles using predefined inclusion criteria. Disagreements were resolved by consensus. Two investigators (CC and CH) independently extracted the data using a standardized data extraction from. Disagreements were again resolved by consensus.

We collected the following data: study design, outcome of interest (death, relapse), number of patients and number of events, survival curves estimate, regression method to estimate the hazard rate (Cox PH model or parametric survival model), methods for comparing the survival curves, the shape of the survival curves, variable selection, model building strategy, censoring description, length of follow-up, sample size calculation, test of interaction between variables, test for time dependent covariates, test for proportionality assumption and test for goodness-of-fit.

### Analytic criteria

To evaluate the quality of reporting survival analyses, we used the following list of criteria for the proper use and description of the survival analyses.

1. *Sample size:* We evaluated the methods that the investigators described for sample size calculation. In addition, in the studies that used multiple regression analysis we evaluated the number of the events and number of covariates in order to estimate the adequacy of power. According to Peduzzi et al., approximately ten events per covariate is appropriate in PH regression analysis [8].
2. *Censoring description:* We evaluated the description of censoring and whether the investigators reported this adequately, inadequately or there was no mention.
3. *Survival curves:* We evaluated the statistical methods used for generating survival curves. For the comparison of survival between the groups, we documented the reported methods (log-rank test or Wilcoxon test). We also noted the shape of the survival curves (evenly separated or crossing survival curves).
4. *Statistical significance:* The statistical test used to evaluate the difference between two survival curves is determined using a log-rank test or weighted log-rank test (e.g. Wilcoxon test). The null hypothesis of the test is that there is no difference between the two survival curves. We documented the statistical test reported in the articles.
5. *Regression model:* The statistical methods used for survival regression analysis were evaluated. We were interested in the regression model that the investigators used for calculating the hazard ratio (e.g. Cox PH model, extended Cox PH model or parametric survival model). We were also interested in the other regression models (time-dependent variable, competing risk analysis or repeated event analysis). In addition, the test for interaction of the variable was checked. For the studies that used multivariate regression, we assessed the description of variable selection and the strategy used for model building.
6. *Check for the PH assumption:* We assessed the test for PH assumption described in the articles. The assessment included methods used for checking PH assumption (graphical approach or the goodness-of-fit testing approach).
7. *Model checking:* We evaluated whether the investigators assessed for goodness-of-fit measures. The residual-based diagnostics were also assessed (martingale residuals, Cox-Snell residuals, Schoenfeld residuals or deviance residuals).

## Results

### Study characteristics

**Fig. 1:**
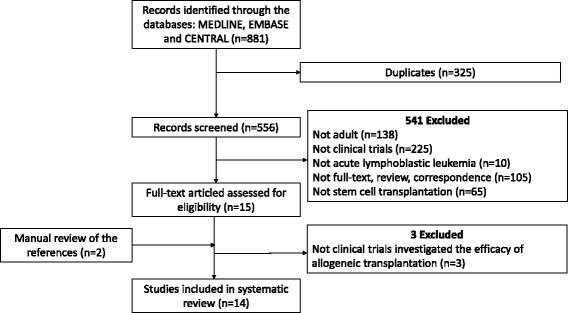
Flow diagram

A total of 881 citations were identified by the systematic search strategy. Of these, 325 studies were duplicates. After screening of the titles and abstracts using predefined inclusion criteria, 541 studies were excluded. The reasons for exclusion are summarised in Fig. 1. Of these, we identified 15 potential studies for full-text review. Two studies were identified following manual review of the references. We excluded three studies due to no clinical trials comparing allo-SCT with other treatments. Thus, 14 studies [9–22] were included in our systematic review.

The study characteristics are summarised in Table 1. All of the studies were clinical trials. Patients were randomized to receive either allo-SCT or other treatments (autologous SCT or consolidation chemotherapy). Patients were allocated to undergo allo-SCT if the patient had a human leukocyte antigen (HLA) matched sibling donor, otherwise, the patient received autologous SCT or consolidation chemotherapy according to the study protocols. The median follow-up ranged from 59 to 114 months. The time-to-event outcomes in the included studies were overall survival and disease-free survival.

**Table 1:** Study characteristics

Study	Sample size		Median Follow-up (months)	Outcome	Design
	Allo-SCT	Non-allo-SCT			
Attal [9]	41	64	30	DFS	Randomized trial
Bernasconi [10]	11	29	48	DFS	Clinical trial
Cornelissen [11]	96	161	65	OS, DFS	Clinical trial
De Witte [12]	30	33	60	OS, DFS	Clinical trial
Fielding [13]	81	77	98	OS, DFS	Clinical trial
Goldstone [14]	443	588	59	OS, DFS	Clinical trial
Hunault [15]	41	106	61	OS, DFS	Randomized^¶^ trial
Ifrah [16]	18	32	60	DFS	Clinical trial
Labar [17]	68	116	114	OS, DFS	Clinical trial
Ribera [18]	84	98	70	OS, DFS	Clinical trial
Sebban [19]	116	141	62	OS, DFS	Clinical trial
Thomas [21]	100	159	62	OS, DFS	Clinical trial
Takeuchi [20]	34	108	63	OS, DFS	Clinical trial
Ueda [22]	17	40	62	OS, DFS	Clinical trial

Genetically randomization

*Abbreviation*: *Allo-SCT* allogeneic-stem cell transplantation, *DFS* disease free survival, *OS* overall survival

### Analytic criteria

1. *Sample size*
  Sample size estimation was described in 4 of 14 studies (Table 2). The proportion of events per total patients ranged from 36 to 78 %. With respect to the sample size and number of covariates assessed in the regression analysis, only four studies reported the list of covariate assessed in the Cox regression model. Of these, two studies obtained more than ten events-per-covariate (event-per-covariate 20.4 and 23.2, respectively) [11, 13]. However, the other two studies had an event-per-covariate 8.3 [20] and 3.8 [22].

**Table 2:** Reporting survival analyses in the included studies

Study	Sample size estimation	Event/total	Censoring	Survival curve	Shape of KM-curve	Significance	Regression model	Variable selection	Goodness of fit or PH assumption test
Attal [9]	Yes	86/135 (relapse)	NR	KM method, log-rank test	Crossing, overlapping	S	Cox-PH model	Informal	NR
Bernasconi [10]	NR	NR	NR	KM method, log-rank test	Crossing	NS	N/A	N/A	NR
Cornelissen [11]	NR	102/257 (death), 113/257 (relapse)	Yes	KM method, log-rank test	Crossing	NS	Cox-PH model, interaction, competing risk	Informal	NR
De Witte [12]	NR	32/66 (death)	NR	KM method, log-rank test, Wilcoxon test	Crossing, overlapping	S	N/A	N/A	NR
Fielding [13]	NR	116/165 (death)	NR	KM method, log-rank test	Crossing	NS	N/A	N/A	NR
Goldstone [14]	NR	531/1031 (death)	Yes	KM method, log-rank test	Crossing, overlapping	NS	Cox-PH model	N/A	NR
Hunault [15]	Yes	67/147 (death)	NR	KM method, log-rank test	Crossing	S	Cox-PH model	Informal	NR
Ifrah [16]	Yes	37/50 (relapse)	NR	KM method, log-rank test	Curves were plotted in two different graphs	N/A	Cox-PH model	Informal	NR
Labar [17]	NR	245/340 (death), 131/340 (relapse)	Yes	KM method, log-rank test	Crossing, overlapping	NS	Cox-PH model	Informal	NR
Ribera [18]	Yes	144/182 (death), 88/182 (relapse)	Yes	KM method, log-rank test	Crossing	NS	Cox-PH model	Informal	NR
Sebban [19]	NR	146/255 (death)	NR	KM method, log-rank test	Crossing	NS	N/A	N/A	NR
Thomas [21]	NR	177/259 (relapse)	NR	KM method, log-rank test	Unevenly separate	S	Cox-PH model	Informal	NR
Takeuchi [20]	NR	83/142 (death)	Yes	KM method, log-rank test	Crossing, overlapping	NS	Cox-PH model	Informal	NR
Ueda [22]	NR	38/57 (death)	Yes	KM method, log-rank test, Wilcoxon test	Evenly separate	S	Cox-PH model	Informal	NR

*Abbreviation*: *PH* proportional hazard, *NR* not reported, *N/A* not applicable; S, statistical significance; NS, non-statistical significance
2. *Censoring description*
  There were six studies that provided the censoring description.
3. *Survival curves*
  All studies reported survival curves using the KM method. The comparisons between the groups were investigated using a log-rank test in all studies (two studies used both log-rank test and Wilcoxon test). With regards to the shape of the survival curves, 11 studies reported crossing survival curves [9–15, 17–20] whereas one study reported unevenly separate survival curves [21] and one study reported evenly separated survival curves [22]. The overlapping survival curves were observed in five studies [9, 12, 14, 17, 20]. We were not able to compare survival curves in one study where the graphs were plotted in the separately [16].
4. *Statistical significance*
  All of the studies reported the statistical test used to measure the difference between survival curves. Of these, five studies reported statistical significance for the treatment effect between groups. However, eight studies reported non-statistical significance (one study did not report).
5. *Regression model*
  The regression model was incorporated in 10 of 14 studies. All ten studies used the Cox PH model [9, 11, 14–18, 20–22]. There was no parametric survival analysis used in the included studies. One study mentioned the test for interaction and competing risk analysis [11]. None of the studies described variable selection. Only one study mentioned the strategy used for model building [18].
6. *Check for the PH assumption*
  In studies that used Cox PH model, PH assumption checking was not mentioned in any of the studies that used Cox PH model.
7. *Model checking*
  The summary measures of the regression diagnostic and goodness-of-fit were not mentioned in any of the studies.

## Discussion

Our study demonstrates that survival analyses have been used extensively in the landmark trials evaluating all-SCT in adult patients with acute ALL. However, the majority of the trials poorly reported their statistical methods and results. Sample size estimation and censoring description were not routinely described. Almost all the presented survival curves crossed. Moreover, the Cox assumption was not assessed even if the investigators used the Cox PH model. In addition, goodness-of-fit or regression residual analysis were lacking in all of the trials.

Regarding the sample size estimation, according to Consolidated Standards of Reporting Trials (CONSORT), it is important that the authors indicate how sample size was determined [23]. The intent of the sample size estimation is to ensure that a particular study has sufficient statistical power to detect a difference in the treatment effect between groups. Our review demonstrates that only 4 of 14 (29 %) trials described a sample size estimation. With respect to the regression analysis, only four studies provided a full list of covariates. Of these, only two studies appeared to be sufficiently powered (event-to-covariate ratio more than 10).

In survival analysis, patients who do not experience the relevant outcome over the study period, patients who are lost to follow-up during the study period and patients who withdraw from the study are censored. There are three assumptions regarding censorship in survival analysis: independent, random and non-informative [4]. Thus, the description of censorship is an important aspect to report in publication. However, only 6 of 14 (43 %) trials described their censoring. More importantly, if relapse is the outcome of interest in these studies, patients who die from any cause will be censored. In this circumstance, censoring may be considered informative because patients may die from disease progression or treatment-related causes. Consequently, the results may change based on different censoring descriptions. Providing a definition of censorship is a critical component to reporting these trials in the literature.

All of the studies utilized survival curves. Not surprisingly, crossing survival curves were found in 10 of 14 studies. Allo-SCT is considered the most potent post remission therapy in adult ALL [24]. In long-term follow-up studies, the patients who underwent allo-SCT had a lower relapse rate due to a graft-versus-leukemia effect [11]. However, these patients had a higher early mortality rate from the toxicity of myeloablative chemotherapy when compared with patients who received autologous SCT or consolidation chemotherapy [11, 13]. Therefore, survival curves comparing these two treatments may be expected to cross at some point. Early death from treatment-related complications (commonly found in allo-SCT) and late death from relapsed disease (commonly found in autologous SCT) should be taken into the account in the treatment of ALL. Crossing survival curves make the interpretation of the treatment effects from the interventions much more complicated.

The log-rank test is the most common method used to compare the difference between survival curves based on the chi-square test [25]. It is important to note that the log-rank test may be invalid if the survival curves cross because of an increase of the probability of type II error. Moreover, the log-rank test may lose power in the circumstance of crossing survival curves [26]. Our study reveals that, among ten analyses with crossed survival curves, eight were non-statistically significant and two were statistically significant. We found that five studies had overlapping survival curves that might be explainable for insignificant findings of the interventions. It was difficult to make a conclusion on the rest of the studies based on the log-rank test of crossing over survival curves.

Strategies have been proposed to overcome the limitation of the log-rank test when the survival curves cross. The authors may consider analysing the survival curves at a fixed point in time [27]. Another alternative includes using a weighted log-rank (Harrington-Fleming) test which gives more weight to the later events [28]. Other weighted log-rank tests that may be useful are the methods developed by Gill et al. or Pepe and Fleming [29, 30]. Li et al. recently published a simulation study which investigated several statistical methods in the situation of crossing survival curves. This study showed that adaptive Neyman’s smooth tests and the two-stage procedure provided greater stability and higher power as compared to the other methods [29].

Relapse disease and death are the most common outcomes in the ALL literature. Conventional KM method and Cox proportional hazard model convey no information regarding possible competing risks. Competing risk is an event that modifies the chance of the interested outcome [31]. For example, death from any cause is a competing risk for relapse disease. Using the competing risk analysis is therefore considered to be more appropriate in the treatment with high rate of complications. We observed only one study that used competing risk analysis [11]. We encouraged investigators to incorporate competing risk analysis, at least in the sensitivity analysis.

We found that the Cox PH model was commonly used in the collection of articles in our review. There was substantial inadequacy of the description of variable selection, the strategy used for fitting procedure and test for goodness-of-fit. As mentioned above, sample size estimation related to regression analysis was noted in only four studies. Of these, two studies were found to be underpowered based on low event-per-covariate ratio [8]. We strongly encourage authors to describe the process of variable selection, strategy of model building and provide evidence that the sample size is sufficient for regression analysis.

A lack of PH assumption checking may introduce bias to the regression analysis. Our review shows that none of the studies described an assessment of the PH assumption. The Cox PH model assumes that the hazard ratio for comparing any two groups of predictor variables is constant over time [4]. If this assumption is not met, the Cox PH model is not valid for the analysis. We observed that 11 of 14 (79 %) studies had crossing survival curves. A clear violation of the PH assumption occurs if survival curves cross [32, 33]. Therefore, a hazard ratio should not be used to compare the treatment effect between groups. We suggest that authors check for the PH assumption if the Cox PH model is incorporated in the analysis. When the PH assumption is violated, authors may consider using an alternative regression analysis, such as the extended Cox PH model or parametric survival analysis (Weibull, exponential, log-logistic or lognormal model).

## Conclusions

Our systematic review evaluating reporting methods for survival analysis in adult ALL patients undergoing allo-SCT show significant shortcomings in the use and reporting of survival analysis. Sample size estimation was not routinely described and studies are frequently statistically underpowered. There was a lack of censoring description. Most notably, crossing survival curves were common and none of the studies checked for the PH assumption. Finally, the description of variable selection, fitting procedure and model checking were neglected.

Survival analysis has been used increasingly in medical research studies [6]. We raise awareness of these limitations and encourage authors, reviewers and editors to improve the quality of the use and reporting survival analysis in the literature.

## Abbreviations

ALL, acute lymphoblastic leukemia; CONSORT, Consolidated Standards of Reporting Trials; HLA, human leukocyte antigen; HR, hazard ratio; KM, Kaplan-Meier; PH, proportional hazard; SCT, stem cell transplantation

## References

[1] Clark TG, Bradburn MJ, Love SB, Altman DG (2003). Survival analysis part I: basic concepts and first analyses. Br J Cancer.

[2] Peto R, Pike MC, Armitage P, Breslow NE, Cox DR, Howard SV, Mantel N, McPherson K, Peto J, Smith PG (1977). Design and analysis of randomized clinical trials requiring prolonged observation of each patient. II. Analysis and examples. Br J Cancer.

[3] Cox DR (1972). Regression models and life-tables. J R Stat Soc.

[4] Kleinbaum DG, MKlein M (2012). Survival analysis: A self-lerning text, thrid edition.

[5] Bradburn MJ, Clark TG, Love SB, Altman DG (2003). Survival analysis part II: multivariate data analysis--an introduction to concepts and methods. Br J Cancer.

[6] Abraira V, Muriel A, Emparanza JI, Pijoan JI, Royuela A, Plana MN, Cano A, Urreta I, Zamora J (2013). Reporting quality of survival analyses in medical journals still needs improvement. A minimal requirements proposal. J Clin Epidemiol.

[7] Ribera JM (2011). Allogeneic stem cell transplantation for adult acute lymphoblastic leukemia: when and how. Haematologica.

[8] Peduzzi P, Concato J, Feinstein AR, Holford TR (1995). Importance of events per independent variable in proportional hazards regression analysis. II. Accuracy and precision of regression estimates. J Clin Epidemiol.

[9] Attal M, Blaise D, Marit G, Payen C, Michallet M, Vernant JP, Sauvage C, Troussard X, Nedellec G, Pico J (1995). Consolidation treatment of adult acute lymphoblastic leukemia: a prospective, randomized trial comparing allogeneic versus autologous bone marrow transplantation and testing the impact of recombinant interleukin-2 after autologous bone marrow transplantation. BGMT Group. Blood.

[10] Bernasconi C, Lazzarino M, Morra E, Alessandrino EP, Pagnucco G, Resegotti L, Locatelli F, Ficarra F, Bacigalupo A, Carella AM (1992). Early intensification followed by allo-BMT or auto-BMT or a second intensification in adult ALL: a randomized multicenter study. Leukemia.

[11] Cornelissen JJ, van der Holt B, Verhoef GE, van’t Veer MB, van Oers MH, Schouten HC, Ossenkoppele G, Sonneveld P, Maertens J, van Marwijk KM (2009). Myeloablative allogeneic versus autologous stem cell transplantation in adult patients with acute lymphoblastic leukemia in first remission: a prospective sibling donor versus no-donor comparison. Blood.

[12] De Witte T, Awwad B, Boezeman J, Schattenberg A, Muus P, Raemaekers J, Preijers F, Strijckmans P, Haanen C (1994). Role of allogenic bone marrow transplantation in adolescent or adult patients with acute lymphoblastic leukaemia or lymphoblastic lymphoma in first remission. Bone Marrow Transplant.

[13] Fielding AK, Rowe JM, Richards SM, Buck G, Moorman AV, Durrant IJ, Marks DI, McMillan AK, Litzow MR, Lazarus HM, Foroni L, Dewald G, Franklin IM, Luger SM, Paietta E, Wiernik PH, Tallman MS, Goldstone AH (2009). Prospective outcome data on 267 unselected adult patients with Philadelphia chromosome-positive acute lymphoblastic leukemia confirms superiority of allogeneic transplantation over chemotherapy in the pre-imatinib era: results from the International ALL Trial MRC UKALLXII/ECOG2993. Blood.

[14] Goldstone AH, Richards SM, Lazarus HM, Tallman MS, Buck G, Fielding AK, Burnett AK, Chopra R, Wiernik PH, Foroni L, Paietta E, Litzow MR, Marks DI, Durrant J, McMillan A, Franklin IM, Luger S, Ciobanu N, Rowe JM (2008). In adults with standard-risk acute lymphoblastic leukemia, the greatest benefit is achieved from a matched sibling allogeneic transplantation in first complete remission, and an autologous transplantation is less effective than conventional consolidation/maintenance chemotherapy in all patients: final results of the International ALL Trial (MRC UKALL XII/ECOG E2993). Blood.

[15] Hunault M, Harousseau JL, Delain M, Truchan-Graczyk M, Cahn JY, Witz F, Lamy T, Pignon B, Jouet JP, Garidi R, Caillot D, Berthou C, Guyotat D, Sadoun A, Sotto JJ, Lioure B, Casassus P, Solal-Celigny P, Stalnikiewicz L, Audhuy B, Blanchet O, Baranger L, Bene MC, Ifrah N, Goelams Group (2004). Better outcome of adult acute lymphoblastic leukemia after early genoidentical allogeneic bone marrow transplantation (BMT) than after late high-dose therapy and autologous BMT: a GOELAMS trial. Blood.

[16] Ifrah N, Witz F, Jouet JP, Francois S, Lamy T, Linassier C, Pignon B, Berthou C, Guyotat D, Cahn JY, Harousseau JL (1999). Intensive short term therapy with granulocyte-macrophage-colony stimulating factor support, similar to therapy for acute myeloblastic leukemia, does not improve overall results for adults with acute lymphoblastic leukemia. GOELAMS Group. Cancer.

[17] Labar B, Suciu S, Zittoun R, Muus P, Marie JP, Fillet G, Peetermans M, Stryckmans P, Willemze R, Feremans W, Jaksic B, Bourhis JH, Burghouts JP, Witte T (2004). Allogeneic stem cell transplantation in acute lymphoblastic leukemia and non-Hodgkin’s lymphoma for patients < or = 50 years old in first complete remission: results of the EORTC ALL-3 trial. Haematologica.

[18] Ribera JM, Oriol A, Bethencourt C, Parody R, Hernandez-Rivas JM, Moreno MJ, del Potro E, Torm M, Rivas C, Besalduch J, Sanz MA, Ortega JJ, Pethema Group Spain. Comparison of intensive chemotherapy, allogeneic or autologous stem cell transplantation as post-remission treatment for adult patients with high-risk acute lymphoblastic leukemia. Results of the PETHEMA ALL-93 trial. Haematologica. 2005;90(10):1346–56. 16219571

[19] Sebban C, Lepage E, Vernant JP, Gluckman E, Attal M, Reiffers J, Sutton L, Racadot E, Michallet M, Maraninchi D (1994). Allogeneic bone marrow transplantation in adult acute lymphoblastic leukemia in first complete remission: a comparative study. French group of therapy of adult acute lymphoblastic leukemia. J Clin Oncol.

[20] Takeuchi J, Kyo T, Naito K, Sao H, Takahashi M, Miyawaki S, Kuriyama K, Ohtake S, Yagasaki F, Murakami H, Asou N, Ino T, Okamoto T, Usui N, Nishimura M, Shinagawa K, Fukushima T, Taguchi H, Morii T, Mizuta S, Akiyama H, Nakamura Y, Ohshima T, Ohno R (2002). Induction therapy by frequent administration of doxorubicin with four other drugs, followed by intensive consolidation and maintenance therapy for adult acute lymphoblastic leukemia: the JALSG-ALL93 study. Leukemia.

[21] Thomas X, Boiron JM, Huguet F, Dombret H, Bradstock K, Vey N, Kovacsovics T, Delannoy A, Fegueux N, Fenaux P, Stamatoullas A, Vernant JP, Tournilhac O, Buzyn A, Reman O, Charrin C, Boucheix C, Gabert J, Lheritier V, Fiere D (2004). Outcome of treatment in adults with acute lymphoblastic leukemia: analysis of the LALA-94 trial. J Clin Oncol.

[22] Ueda T, Miyawaki S, Asou N, Kuraishi Y, Hiraoka A, Kuriyama K, Minami S, Ohshima T, Ino T, Tamura J, Kanamaru A, Nishikawa K, Tanimoto M, Oh H, Saito K, Nagata K, Naoe T, Yamada O, Urasaki Y, Sakura T, Ohno R (1998). Response-oriented individualized induction therapy with six drugs followed by four courses of intensive consolidation, 1 year maintenance and intensification therapy: the ALL90 study of the Japan adult leukemia study group. [erratum appears in Int J hematol 1998 Dec;68(4):i-ii]. Int J Hematol.

[23] Moher D, Hopewell S, Schulz KF, Montori V, Gotzsche PC, Devereaux PJ, Elbourne D, Egger M, Altman DG (2010). CONSORT 2010 explanation and elaboration: updated guidelines for reporting parallel group randomised trials. BMJ.

[24] Goldstone AH, Rowe JM. Transplantation in adult ALL. Hematology Am Soc Hematol Educ Program 2009:593–601. 10.1182/asheducation-2009.1.593 20008244

[25] Rich JT, Neely JG, Paniello RC, Voelker CC, Nussenbaum B, Wang EW (2010). A practical guide to understanding Kaplan-Meier curves. Otolaryngol Head Neck Surg.

[26] Liu K, Qiu P, Sheng J (2007). Comparing two crossing hazard rates by Cox proportional hazards modelling. Stat Med.

[27] Klein JP, Logan B, Harhoff M, Andersen PK (2007). Analyzing survival curves at a fixed point in time. Stat Med.

[28] Logan BR, Klein JP, Zhang MJ (2008). Comparing treatments in the presence of crossing survival curves: an application to bone marrow transplantation. Biometrics.

[29] Li H, Han D, Hou Y, Chen H, Chen Z (2015). Statistical inference methods for two crossing survival curves: a comparison of methods. PLoS One.

[30] Pepe MS, Fleming TR (1989). Weighted Kaplan-Meier statistics: a class of distance tests for censored survival data. Biometrics.

[31] Satagopan JM, Ben-Porat L, Berwick M, Robson M, Kutler D, Auerbach AD (2004). A note on competing risks in survival data analysis. Br J Cancer.

[32] Bouliotis G, Billingham L (2011). Crossing survival curves: alternatives to the log-rank test. Trials.

[33] Seruga B, Amir E, Tannock I (2009). Treatment of lung cancer. N Engl J Med.

